# Supplementary material for: Biofouling of inlet pipes affects water quality in running seawater aquaria and compromises sponge cell proliferation
Source: PeerJ. 2015 Dec 7;3:e1430. doi: 10.7717/peerj.1430 (PMC4675111; doi:10.7717/peerj.1430)

ESM Figure S1. Detailled pictures of biofouling communities within the old inlet pipe. The first 14 m from the entrance of the inlet pipe are shown. No biofouling communities were found after the initial 12 m. Water samples inside the pipe were taken from areas of the pipe outlined in red and marked by the red arrows (0, 3, 6, and 12 m).

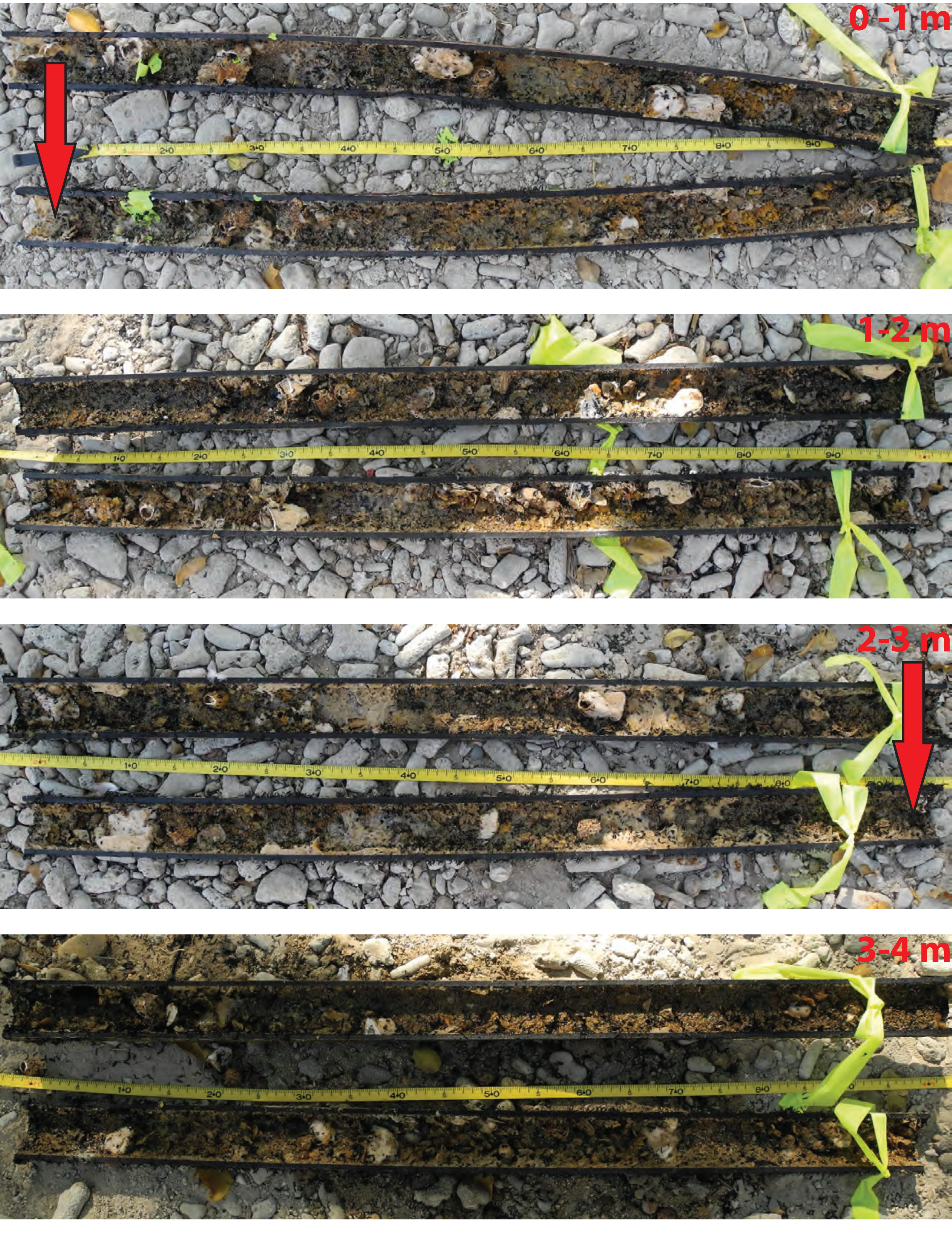

Supplement: Figure S1 [file peerj-03-1430-s001.pdf]
