# Supplementary figures and images for: Biofouling of inlet pipes affects water quality in running seawater aquaria and compromises sponge cell proliferation
Source: PeerJ. 2015 Dec 7;3:e1430. doi: 10.7717/peerj.1430 (PMC4675111; doi:10.7717/peerj.1430)

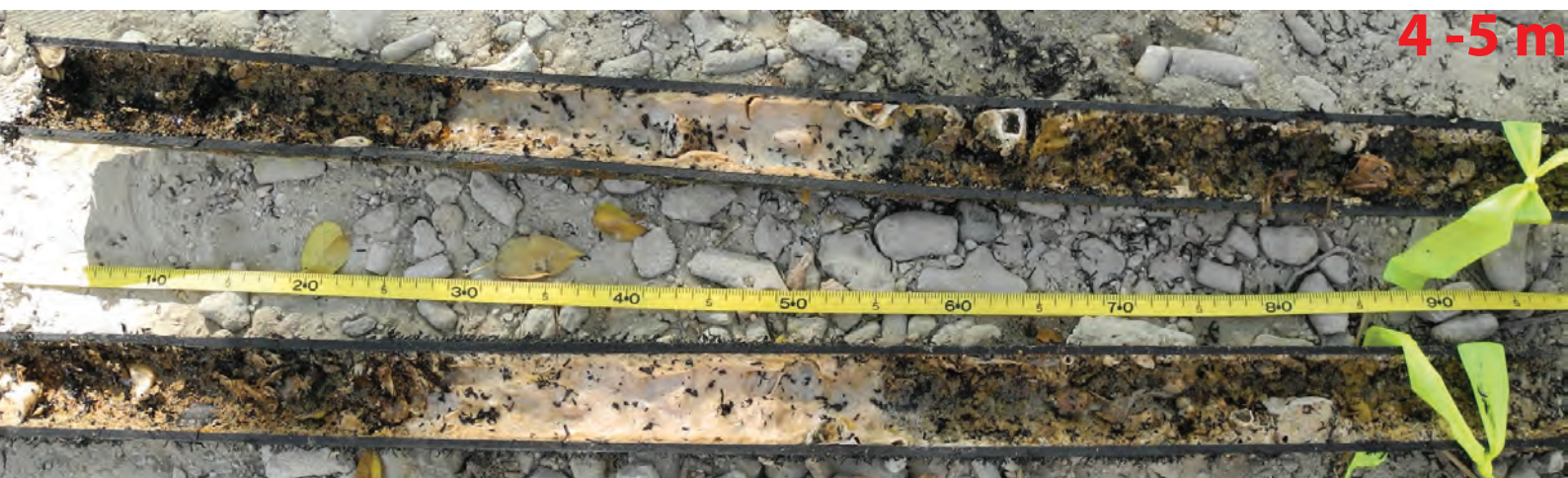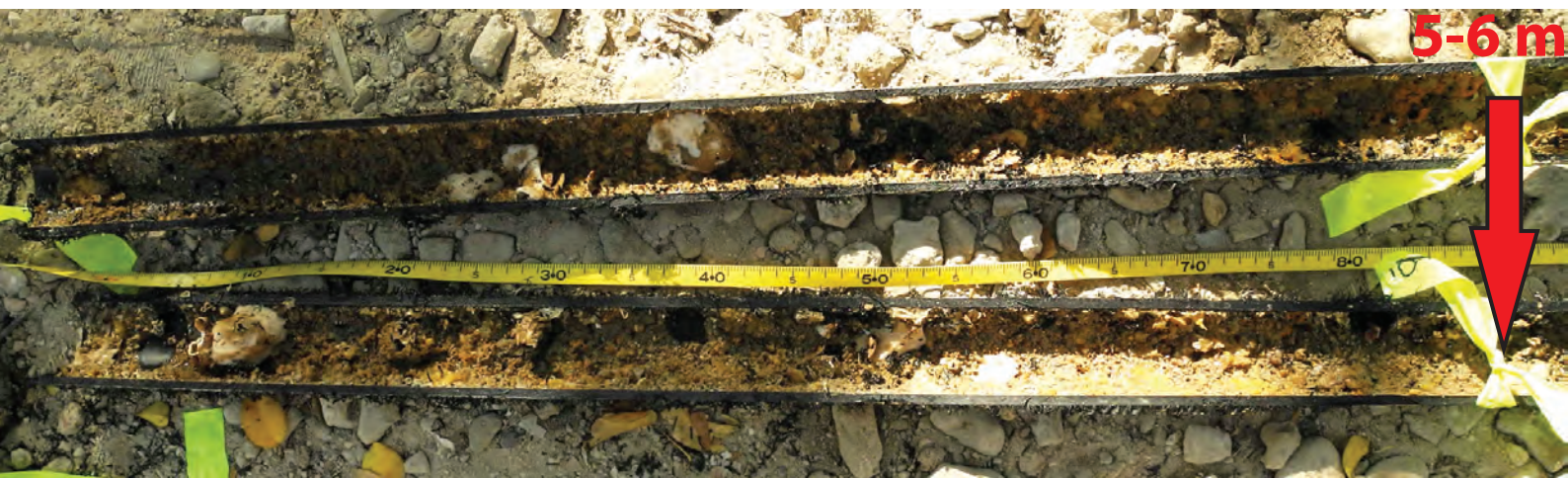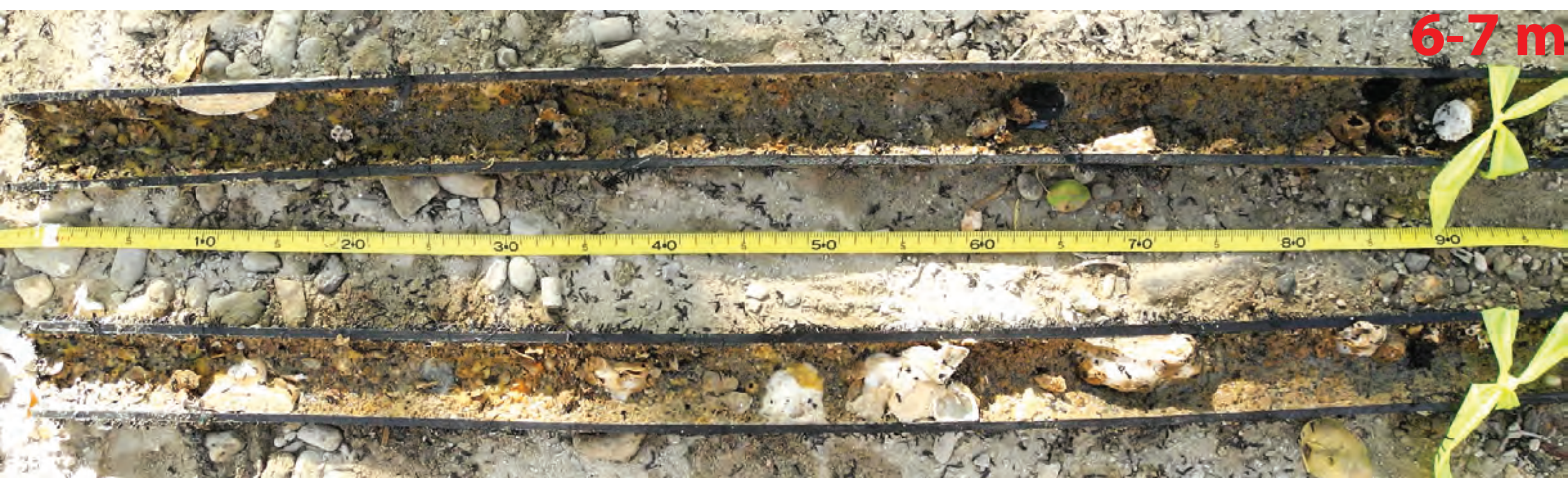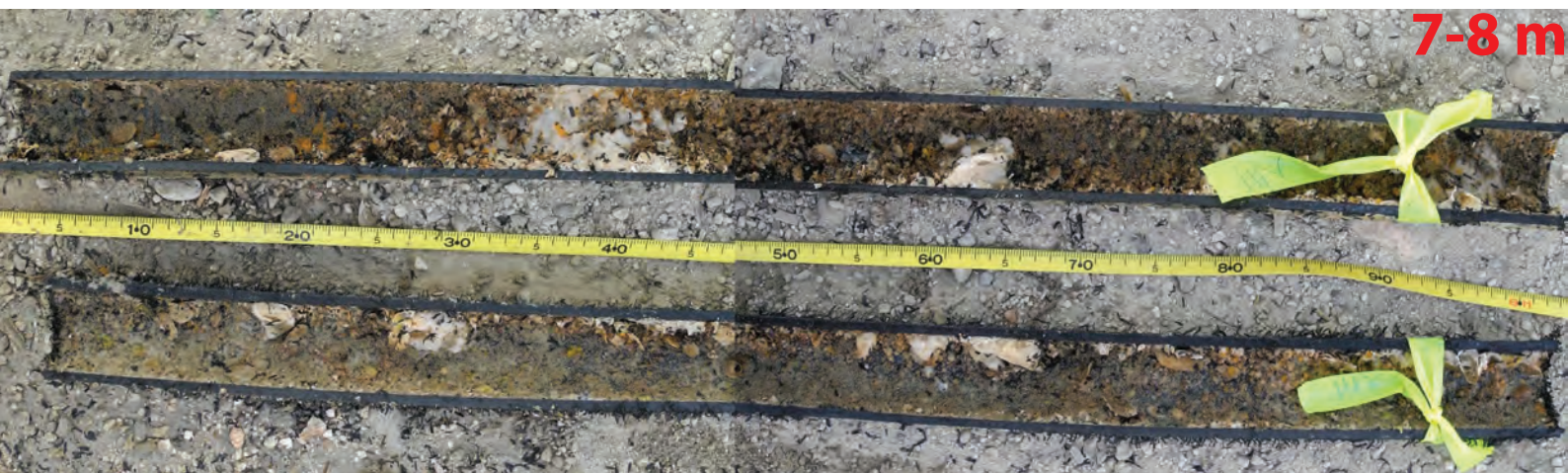

Supplement: Supplemental Information 2 [file peerj-03-1430-s002.pdf]

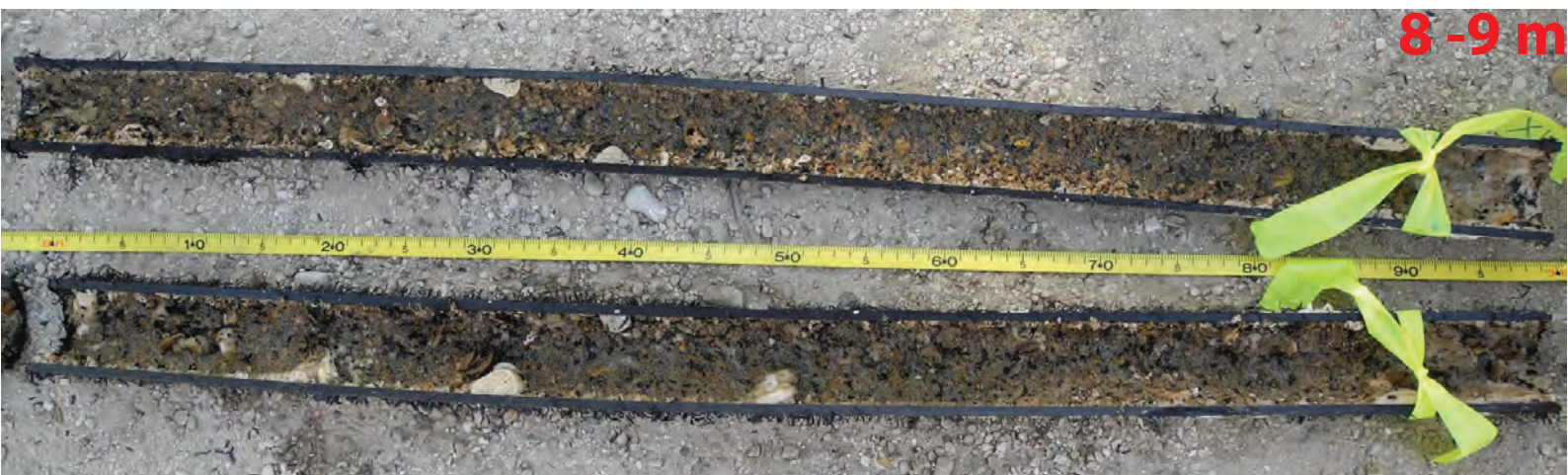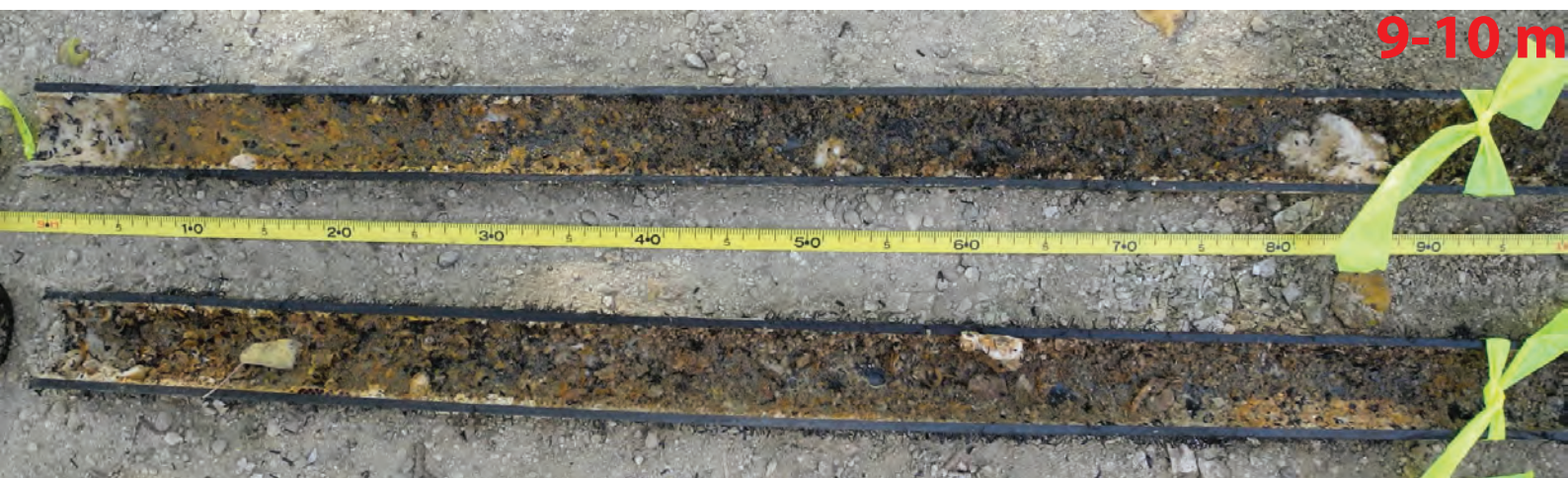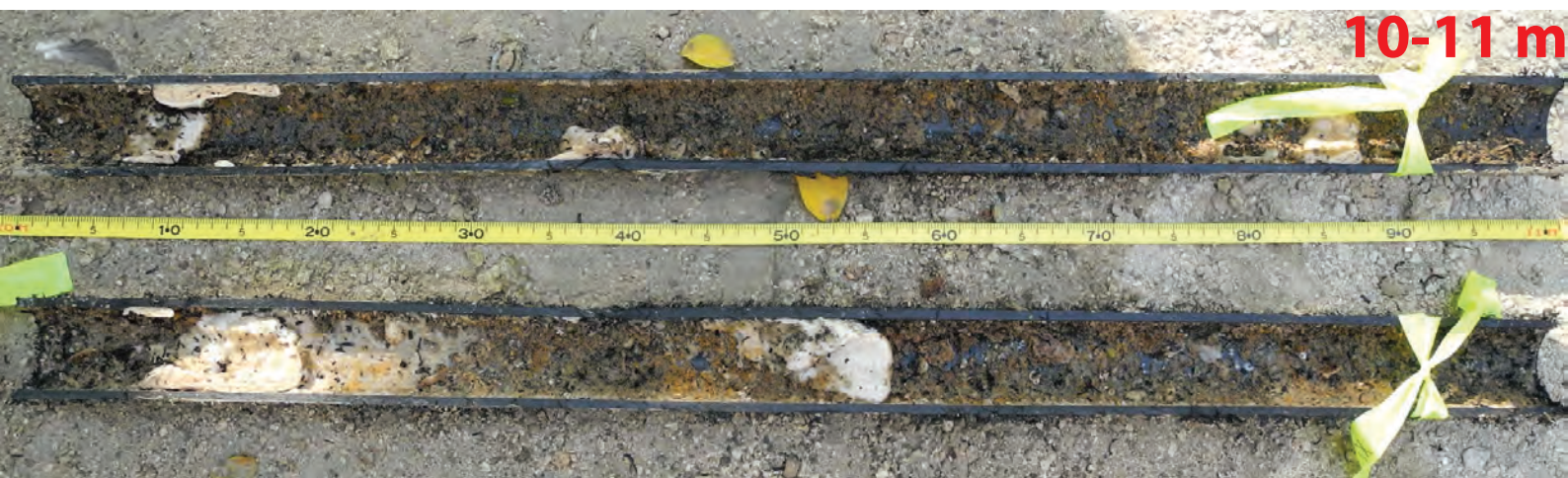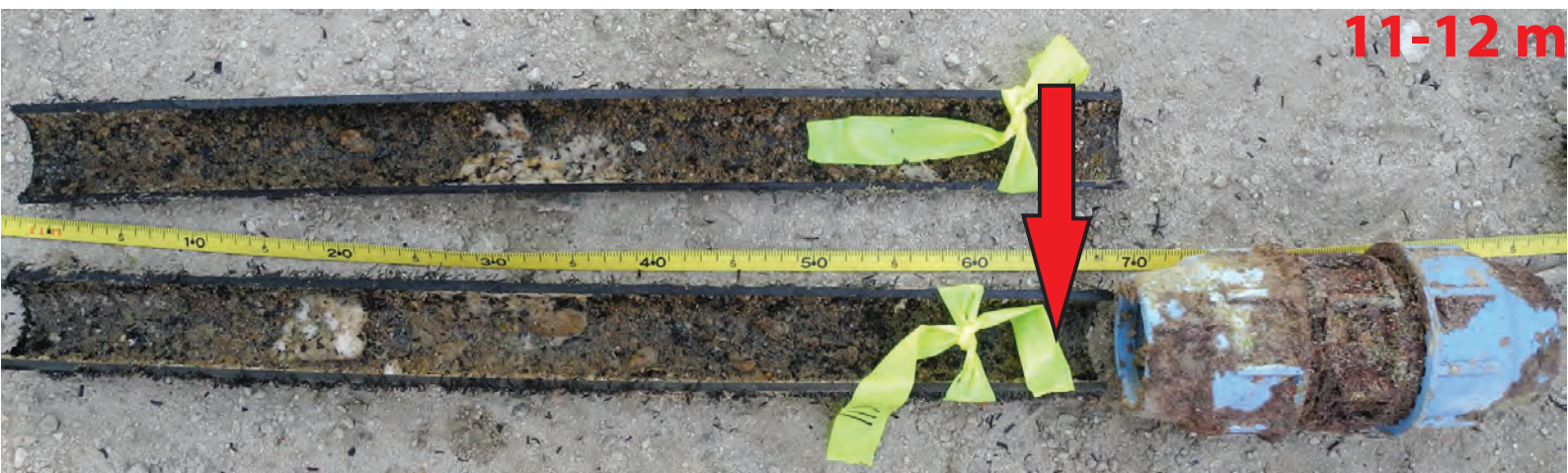

Supplement: Supplemental Information 3 [file peerj-03-1430-s003.pdf]

12-13 m

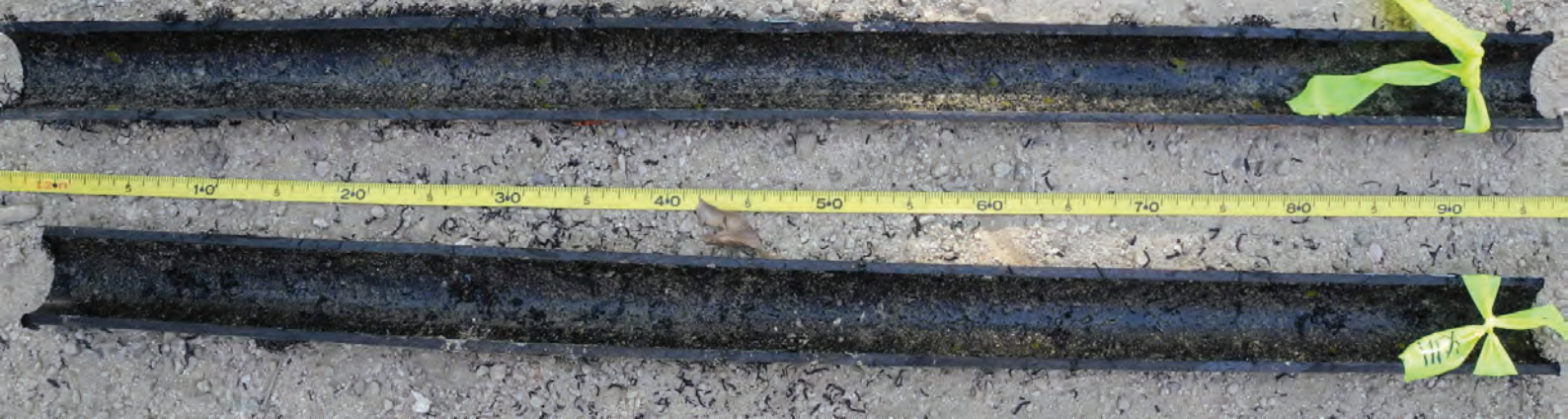

13-14 m

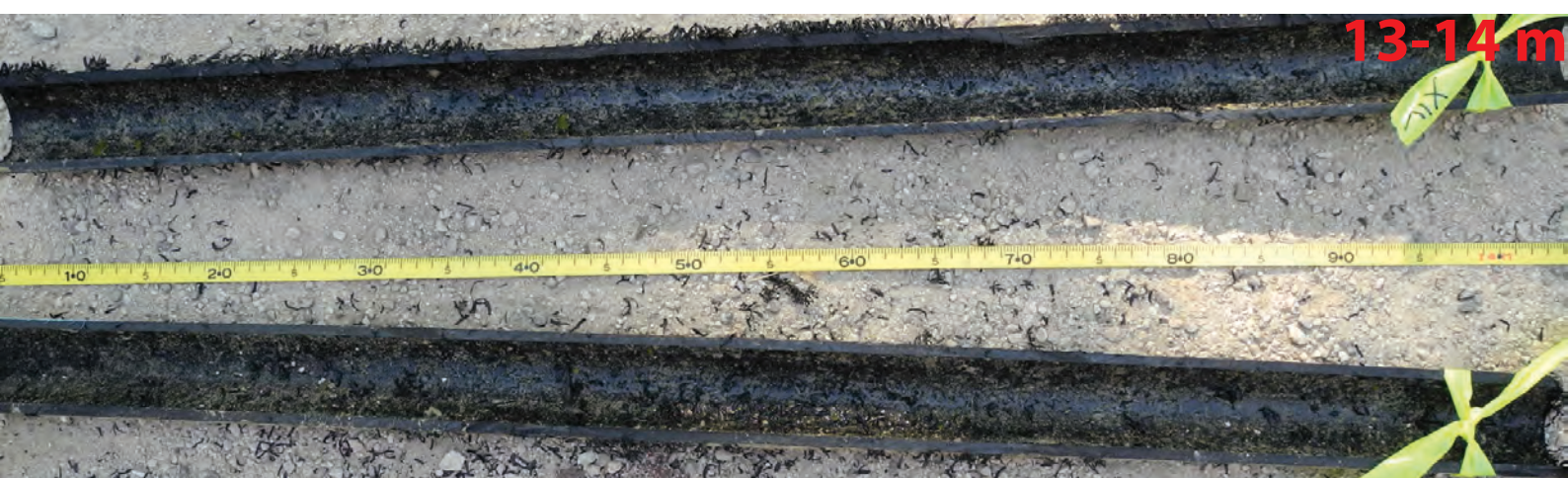

Supplement: Supplemental Information 4 [file peerj-03-1430-s004.pdf]
