# Supplementary material for: Biofouling of inlet pipes affects water quality in running seawater aquaria and compromises sponge cell proliferation
Source: PeerJ. 2015 Dec 7;3:e1430. doi: 10.7717/peerj.1430 (PMC4675111; doi:10.7717/peerj.1430)
Supplement: Supplemental Information 5 [file peerj-03-1430-s006.docx]

data<- read.csv('S2.csv', header = TRUE, sep=";")

data

#bacteria

mexp <- lm(log(conc) ~ pipe, data=data)

summary(mexp)

onlynew = data[data$pipe=='new',]

mexpnew <- lm(log(conc) ~ distance, data=onlynew)

summary(mexpnew)

onlyold= data[data$pipe=='old',]

mexpold <- lm(log(conc) ~ distance, data=onlyold)

summary(mexpold)

#nh4

m2 <- lm(nh4 ~ pipe, data=data)

anova(m2)

m3 <- lm(nh4 ~ distance, data=onlynew)

anova(m3)

m4 <- lm(nh4 ~ distance, data=onlyold)

anova(m4)

#NO2

m5 <- lm(no2 ~ pipe, data=data)

anova(m5)

m6 <- lm(no2 ~ distance, data=onlynew)

anova(m6)

m7 <- lm(no2 ~ distance, data=onlyold)

anova(m7)

# n03

m8 <- lm(no3 ~ pipe, data=data)

anova(m8)

m9 <- lm(no3 ~ distance, data=onlynew)

anova(m9)

m10 <- lm(no3 ~ distance, data=onlyold)

anova(m10)

# PO4

m11 <- lm(po4 ~ pipe, data=data)

anova(m11)

m12 <- lm(po4 ~ distance, data=onlynew)

anova(m12)

m13 <- lm(po4 ~ distance, data=onlyold)

anova(m13)

data2<- read.csv('S4.csv', header = TRUE, sep=";")

data2

#bact entrance

m14 <- lm(conc ~ pipe, data=data2)

anova(m14)

# PO4 entrance

m15 <- lm(po4 ~ pipe, data=data2)

anova(m15)

# NO2 entrance

m16 <- lm(no2 ~ pipe, data=data2)

anova(m16)

# NO3 entrance

m17 <- lm(no3 ~ pipe, data=data2)

anova(m17)

# NH4 entrance

m18 <- lm(nh4 ~ pipe, data=data2)

anova(m18)
